# Assessment of Trends in Non-Restorative and Preventative Dental Treatment Pre- and Post-COVID-19: A Health Informatics Pilot Study

**DOI:** 10.3390/children12030357

**Published:** 2025-03-14

**Authors:** Tanner Gamble, Carter Wilkerson, Cindy Kim, Karl Kingsley, Victoria Sullivan

**Affiliations:** 1 Department of Advanced Education in Pediatric Dentistry, Las Vegas—School of Dental Medicine, University of Nevada, 1700 West Charleston Blvd., Las Vegas, NV 89106, USAvictoria.sullivan@unlv.edu (V.S.); 2 Department of Clinical Sciences, Las Vegas—School of Dental Medicine, University of Nevada, 1700 West Charleston Blvd., Las Vegas, NV 89106, USA; 3 Department of Biomedical Sciences, Las Vegas—School of Dental Medicine, University of Nevada, 1001 Shadow Lane, Las Vegas, NV 89106, USA

**Keywords:** preventive dental treatment, dental sealants, fluoride varnish, silver diamine fluoride, SARS-CoV-2, COVID-19, health informatics

## Abstract

Background/Objectives: The implementation of preventive treatments in pediatric dental care has been a priority in recent years. Understanding the factors that influence the timing and frequency of childhood preventive treatments, such as the impacts of the COVID-19 pandemic, are the focus of many health informatics researchers. Methods: A retrospective study was approved to assess changes in specific preventive treatments at a pediatric dental school clinic (sealants, fluoride varnish, and silver diamine fluoride) in the three years prior to (2017–2019) and following the COVID-19 pandemic (2020–2022). Results: A detailed analysis of these data revealed significant and unexpected shifts in these preventive services, with significant increases in dental sealants from pre- to post-pandemic (35.1%, *p* = 0.012), but corresponding decreases in other preventive treatments, such as the number of fluoride varnish (−37.6%, *p* = 0.011) and SDF treatments (−24.0%, *p* = 0.032), among this patient population. Conclusions: These data suggest that the selective pursuit of particular preventive dental services and treatments rather than others and understanding these shifts might help health informatics and dental public health researchers understand which factors influenced these decisions and behaviors, such as long-term durability and efficacy (sealants) or changing public perceptions of safety (fluoride and SDF).

## 1. Introduction

The use of non-restorative treatments and preventative dental measures have become key components of pediatric dental practice and high priorities for dental public health researchers in recent years [1,2]. The American Dental Association (ADA) and the American Academy of Pediatric Dentistry (AAPD) have developed many guidelines for the application of these treatments and protocols for young children and adolescents used in the management and prevention of one of the most common childhood diseases, dental caries [3,4]. These protocols typically place the most emphasis on the most effective guidelines for the frequency of dental examinations, as well as the most useful and reliable oral treatments and preventive dental services [5,6,7].

The frequency of dental visits and examinations, particularly among pediatric patients, has been a field of growing research interest, as many studies have demonstrated that both the age of first assessment and time intervals between provider visits are both closely associated with specific risk factors for predicting dental and oral health outcomes among this patient population [8,9,10]. Current recommendations suggest that parents initiate dental visits for their children as young as six months of age up to age five years for the primary assessment of risk and the application of oral and dental health preventive measures [11,12]. Furthermore, guidelines suggest that more frequent visits to update risk assessments and, more importantly, to implement preventive measures are associated with clear and significant improvements in patient outcomes, including lower caries experiences, fewer teeth lost to dental decay, and a reduced number of dental extractions due to poor oral health [13,14].

In addition to the timing and frequency of dental visits, dental and public health researchers have also outlined a broad range of preventive measures that have been developed and implemented over time to better address the needs of pediatric populations with different risk factors [15,16]. For example, the use of dental sealants has been proven to be clinically effective at preventing or reducing the incidence of pit and fissure caries among young children, as well as adolescents [17,18]. However, systematic reviews of this evidence have also demonstrated that additional prevention measures may also be needed to complement the use of dental sealants for many pediatric patients with differing risk profiles [19,20].

These additional prevention measures often include fluoride-based toothpastes and mouthwashes, which have been demonstrated to reduce or prevent caries in pediatric patients across many different risk groups [21,22]. Furthermore, many pediatric patients may also experience increased risk factors for a variety of reasons that may be indications for additional fluoride-based treatments, such as topical fluoride therapy (varnish), which has been demonstrated to drastically improve oral health in high-risk children [23,24]. Moreover, pediatric patients with the highest risk with cases of rampant or severe early childhood caries (SECC) may need additional preventive measures and treatments, which might include the use of silver diamine fluoride (SDF)—a treatment that has been demonstrated to be most effective among young children two to eight years of age [25,26].

Despite these efforts to provide preventive dental and oral healthcare treatments, many children and adolescents were negatively impacted by the SARS-CoV-2 (COVID-19) pandemic, which not only reduced clinic capacity but significantly increased delays in patient care and disease prevention-seeking behaviors among many populations across the world [27,28]. Although many novel technologies were developed and implemented, such as the widespread use of telemedicine and teledentistry, these applications could not utilize the most effective prevention measures, which include the use of in-person, provider-based preventive treatments, such as dental sealants, fluoride varnish, and SDF [29,30,31]. In fact, the impact of this pandemic and the effects on pediatric preventive dental care remains a relevant topic for epidemiologists and health informatics researchers, as changes to the frequency and scope of preventive dental visits for children can have lasting impacts on oral health, health seeking and prevention behaviors, and dental disease risk [32,33].

The University of Nevada, Las Vegas—School of Dental Medicine (UNLV-SDM), has been focused on serving the low-income, minority pediatric population in the Las Vegas area with resources, information, education, and the provision of low-cost preventive services for many years [34,35,36]. Initial pilot studies from this institution also evaluated COVID-19 dental protocols for the prevention of oral contamination with the SARS-CoV-2 virus [37,38]. However, due to the lack of information regarding patient visits and preventive services, more research is needed to determine the depth and breadth of the impact of COVID-19 on pediatric patient visits, as well as the number of preventive dental services provided to the local community and how these may have changed over time [39,40]. Based upon this lack of evidence, the primary goal of this study was to evaluate the number of pediatric patient visits and the changes to specific preventive dental services (sealants, fluoride varnish, and SDF) in the years just prior to and following the outbreak of COVID-19 among this pediatric patient population.

## 2. Materials and Methods

### 2.1. Study Review and Approval

Approval for this project was granted by the Institutional Review Board (IRB) and the Office for the Protection of Research Subjects (OPRS) at the University of Nevada, Las Vegas (UNLV), under Protocol #1619329-1 “Retrospective analysis of Oral Health Status of Dental Population”. As this study was conducted as a retrospective review of previously collected data and records with no potential for identified patients or identifiers linked with any record or individual, this study was deemed Exempt Research, according to the Department of Health and Human Services (HHS) Federal Regulation, 45 CFR 46.

As no patients were contacted and individual complete patient records were not accessed in this Exempt Research study of retrospective data, informed consent was also waived according to the HHS Basic Policy for the Protection of Research Subjects, 35.101, which designates Exempt Research as study that does not involve patient contact or prospective data collection from patients. Only summary data of basic demographic information, such as the percentage of males and females and the percentage of racial or ethnic minorities and average ages, were provided to the study authors.

### 2.2. Study Parameters

For the purpose of this study, the population of interest was defined as pediatric patients (under 18 years of age) that were patients of record at the UNLV School of Dental Medicine. The inclusion criteria were pediatric patients of record seen between March 2017 and May 2023. Exclusion criteria included any adult patients (over 18 years of age) and any person not a patient of record at the UNLV dental clinic. The specific interventions evaluated included the application of non-invasive dental sealants in permanent molars and premolars, the application of topical fluoride varnish (5% topical sodium fluoride), and application of silver diamine fluoride or SDF (38% fluoride ion or 44,800 ppm) to any primary tooth on any pediatric patient between March 2017 and May 2023. Summary data regarding the number of pediatric patient treatments for these procedures prior to the SARS-CoV-2 pandemic (March 2017 to March 2020) and the three years following the reopening of the clinic following the COVID-19 pandemic (May 2020 to May 2023) were evaluated for comparison. Independent or predictor variables were defined as treatment date (pre-COVID-19 or post-COVID-19), patient age, and patient sex, with the dependent or outcome variables defined as the number of patients receiving these procedures.

### 2.3. Data Analysis

Summary data regarding the number of preventive dental services and the month they were performed were retrieved by the Institutional Technology (IT) department between the three years prior to the SARS-CoV-2 pandemic (March 2017 to March 2020) and the three years following the reopening of the clinic following the COVID-19 pandemic (May 2020 to May 2023). These data included summary information regarding the application of non-invasive dental sealants on permanent molars and premolars, the application of topical fluoride varnish (5% topical sodium fluoride), and the application of silver diamine fluoride or SDF (38% fluoride ion) to any primary teeth. Additional summary data included information regarding overall percentages by sex (male, female), race or ethnicity (Caucasian, Black, Asian, Hispanic, other), and patient ages (in years).

Summary data were input into Microsoft Excel (Redmond, WA, USA) and graphed for analysis. Descriptive statistics were provided (overall number and percentage) and the analysis and comparisons of categorical data (sex, race or ethnicity) were facilitated using Chi square analysis, which is appropriate for non-parametric data. Changes between the pre- and post-pandemic averages in pediatric patient age were analyzed using two-tailed Student’s *t*-tests, which are appropriate for parametric data analysis. Finally, changes between the number of treatments prior to and following the COVID-19 pandemic were evaluated using Fisher’s exact test.

## 3. Results

The overall pediatric clinic patient population profile was assessed to determine the overall composition of the demographic characteristics (Table 1). These data demonstrated that approximately half of the pediatric patients were males (47.8%) and females (52.5%), which was not significantly different from the overall demographic composition of pediatric males (48.7%) and females (50.3%) in the local population, *p* = 0.3251. However, the analysis of race and ethnicity revealed several significant differences among the clinic population compared with the local population. For example, less than one-fourth (22.8%) of the clinic patients were White or Caucasian and only 3.4% were Asian or Pacific Islander, which is significantly lower than the local population (45.4% and 10.6%, respectively). In addition, the percentage of Hispanic or Latino patients found among the clinic population (59.4%) was much higher than that found among the local population (29.9%), *p* = 0.0001. However, similar percentages of African American (Black) patients (13.2%) and Other (including Native American, 1.2%) were represented within the patient population, which was comparable to the local community (12.4% and 1.7%, respectively). Finally, the average age was approximately 9.04 years, which ranged between 3 months and 18 years.

**Figure 1:**
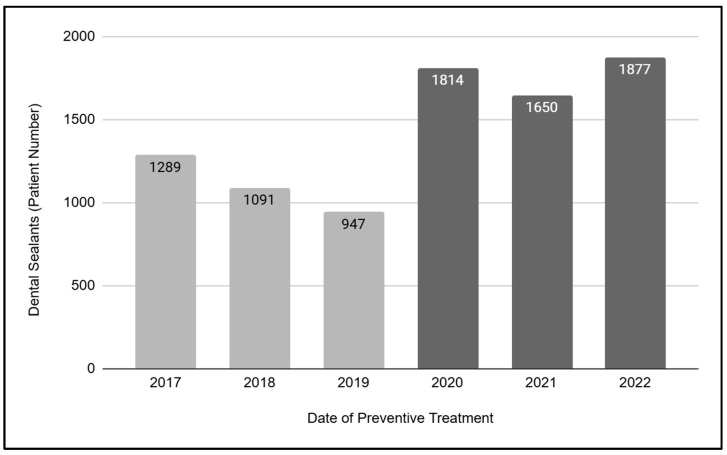
Analysis of dental sealants in pediatric patients (2017 to 2022). A total of N = 8668 dental sealants were applied among pediatric patients between 2017 and 2022. Only *n* = 3327 sealants were applied pre-pandemic (2017, 2018, and 2019; average *n* = 1109), while *n* = 5341 sealants were applied in the three years that followed (2020, 2021, and 2022; average *n* = 1780)—an increase of nearly 60.5%.

To evaluate the changes in preventive services due to the pandemic, an analysis of dental sealants applied in pediatric patients was performed (Figure 1). These data demonstrated that a total of N = 8668 dental sealants were applied in pediatric patients between 2017 and 2022. However, a more detailed analysis revealed that only *n* = 3327 sealants were applied in the three years prior to the SARS-CoV-2 outbreak (2017, 2018, and 2019), while *n* = 5341 sealants were applied in the three years that followed (2020, 2021, and 2022). These data suggest that the overall number and average of sealant applied pre-pandemic (total number *n* = 3327, three-year average *n* =1109) increased by nearly 60.5% in the post-pandemic period (total number *n* = 5341, three-year average *n* = 1780).

**Figure 2:**
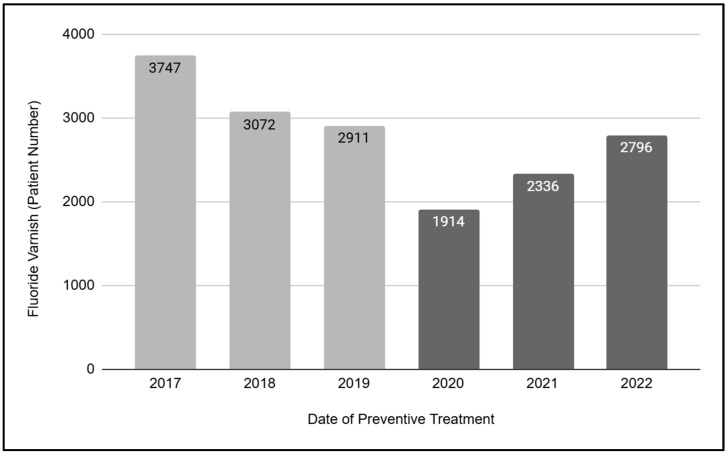
Analysis of fluoride varnish in pediatric patients (2017 to 2022). A total of N = 16,776 fluoride varnish treatments were performed between 2017 and 2022. More treatments were performed in the three years prior to COVID-19, *n* = 9730 (2017, 2018, and 2019; three-year average *n* = 3243.3) than in the three years that followed, *n* = 7046 (2020, 2021, and 2022, three-year average *n* = 2348.7).

To further evaluate the changes in preventive services due to the pandemic, an analysis of fluoride varnish treatments in pediatric patients was performed (Figure 2). These data demonstrated that a total of N = 16,776 fluoride varnish treatments were performed on pediatric patients between 2017 and 2022. More specifically, a much larger number of fluoride treatments were performed during the three years prior to the SARS-CoV-2 outbreak, *n* = 9730 (2017, 2018, and 2019), with significantly fewer treatments in the three years that followed, *n* = 7046 (2020, 2021, and 2022). This analysis revealed that the overall number and average of fluoride treatments performed pre-pandemic (total number *n* = 9730, three-year average *n* = 3242.3) decreased by nearly 37.6% in the post-pandemic period (total number *n* = 7046, three-year average *n* = 2348.7).

**Figure 3:**
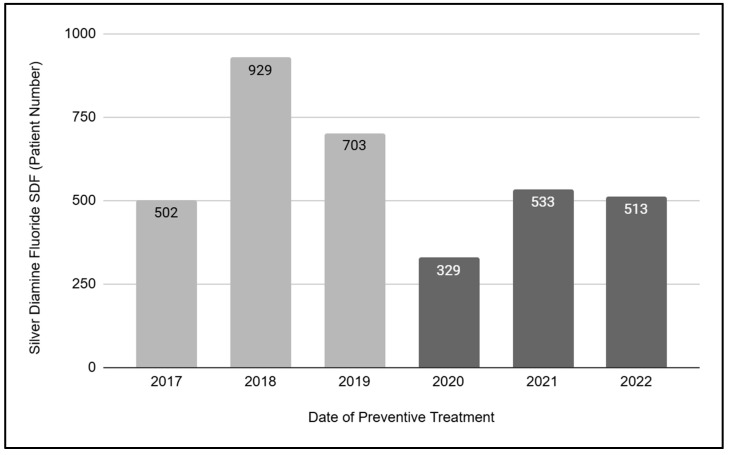
Analysis of silver diamine fluoride (SDF) treatments among pediatric patients (2017 to 2022). A total of N = 3509 pediatric SDF treatments were administered between 2017 and 2022. More SDF treatments were performed during the three years prior to the SARS-CoV-2 outbreak, *n* = 2134 (2017, 2018, and 2019, three-year average *n* = 711.3), with many fewer treatments in the three years that followed, *n* = 1375 (2020, 2021, and 2022, three-year average *n* = 458.3).

The final analysis of preventive dental services was to evaluate silver diamine fluoride (SDF) treatments in the pediatric patients (Figure 3). These data demonstrated that a total of N = 3509 SDF treatments were administered in pediatric patients between the years of 2017 and 2022. A detailed analysis of these data revealed a much greater number of SDF treatments were performed during the three years prior to the SARS-CoV-2 outbreak, *n* = 2134 (2017, 2018, and 2019), with many fewer treatments in the three years that followed, *n* = 1375 (2020, 2021, and 2022). Further, these data demonstrated that the overall number and average number of SDF treatments performed pre-pandemic (total number *n* = 2134, three-year average *n* = 711.3) decreased by nearly 35.6% in the post-pandemic period (total number *n* = 1375, three-year average *n* = 458.3).

To determine the other potential factors that might have influenced the outcomes observed, an analysis of more detailed information regarding the pediatric patients and these preventive treatments was performed (Table 2). The evaluation of dental sealants revealed no significant differences in the age of the patients pre-pandemic and post-pandemic (10.51 and 10.96 years, respectively; *p* = 0.9224) or between the percentages of males and females (approximately 50% and 50%, respectively; *p* = 0.5093). However, a much greater number of patients treated with dental sealants were found in the post-pandemic period compared with the pre-pandemic period (*n* = 6596 vs. *n* = 8913, respectively, *p* = 0.012), which was statistically significant.

The data regarding pediatric patients receiving fluoride varnish revealed an average age lower than those receiving sealants, which did not differ significantly between the pre- and post-pandemic periods (8.55 and 8.97 years, respectively; *p* = 0.231). In addition, the percentage of males and females were nearly equal and did not differ significantly from pre- to post-pandemic, *p* = 0.4911. However, a significant reduction in the number of fluoride varnish treatments was observed, declining from *n* = 9730 to *n* = 7046, or a decrease of 37.6%, *p* = 0.011.

Similarly, an analysis of the data regarding SDF treatments in the pediatric patients demonstrated an even lower average age that did not change significantly over the years before and after the pandemic (6.76 and 6.34 years, respectively; *p* = 0.266). Moreover, no significant differences were found between the percentages of males and females receiving SDF treatment (roughly equal) in the pre- or post-pandemic time frames analyzed, *p* = 0.511. However, a sharp decline in SDF patient treatments was observed in the pre-pandemic period compared with those post-pandemic (*n* = 1809 and *n* = 1375, respectively), which represents an overall decrease of 24.0%, *p* = 0.032.

## 4. Discussion

The overall objective of this study was to use data analytics and health informatics to determine the impact of COVID-19 on the number of pediatric patient visits for specific preventive dental services (sealants, fluoride varnish, and SDF) provided among this pediatric patient population. These data demonstrated significant changes in the number of patient visits for each type of preventive service obtained, supporting other reviews that demonstrated significant shifts in dental and oral healthcare patterns following the COVID-19 pandemic in other patient populations [41,42,43]. However, these data also demonstrated that some preventive dental services (but not others) were more frequently obtained after the onset of the pandemic and did not vary according to either the sex or age of the patient, which may provide some insight into the changes in behavior regarding preventive oral healthcare treatment services sought by parents and their children and the potential identification of factors that might influence and drive these types of healthcare decisions [44,45,46].

More specifically, a much larger number of pediatric patients had dental sealants applied in the post-pandemic period than in the pre-pandemic period—an unusual finding for traditional preventive services that only seems to correspond with other types of post-pandemic increases, specifically in the rates of remote or digital services accessed [47,48,49]. One potential factor that may be driving this move towards preventive services such as dental sealants may be that these are among the most durable and lasting preventive measures, which also have the added benefit that they do not need constant reapplication for clinical efficacy—a strong positive attribute for those parents and pediatric patients who may be limiting their interactions within the post-pandemic dental and oral healthcare system [50,51,52]. In addition, many newer sealants have additional antimicrobial properties that may be more specifically targeted towards reducing cariogenic bacterial loads, which may also be a contributing factor that has driven the increased interest in these preventive measures—particularly among low-income and minority populations that seek to improve protection measures for their children using the most efficient and cost-effective methods [53,54,55].

These data represent a stark shift in the acceptance and parental usage of dental sealants, which had declined in use and popularity almost two decades ago due to the widespread news coverage of the dangers surrounding Bisphenol A (BPA)—an industrial plastic used in the manufacture of personal hygiene products and resins [56,57]. Although the American Dental Association (ADA) and other research has clarified that most dental sealants do not release enough BPA to reach cytotoxic levels through microleakage (less than 0.1 nanograms), more recent formulations prominently feature the BPA-free nature of dental sealants and this awareness and knowledge among dental providers and parents has greatly improved their uptake and use among the general public—particularly among high-risk pediatric patient populations [58,59]. In fact, although there may be residual hesitation on the part of some parents and guardians regarding the safety and efficacy of dental sealants due to the previous controversy surrounding the use of dental sealants containing trace amounts of BPA, this information has largely faded from public view in more recent years and may no longer be a dominant factor in parental decisions regarding the use of dental sealants for their children [60,61,62].

However, the observations made in this study that indicate significant declines in the number of fluoride treatments may indicate the influence of several additional, potentially inter-related factors contributing to these changes in treatments and behaviors in this patient population. More specifically, fluoride varnish is an effective preventive treatment but requires reapplication every three to six months—a stark contrast to the more stable and long-term effects of dental sealants that do not require constant reapplication and additional in-person office visits [63,64]. In addition, fluoride has historically been subject to many widespread concerns regarding safety and potential short- and long-term health risks associated with dental fluoride use among children and adolescents that may have affected parental behaviors since as far back as the mid 1990s [65,66,67]. More recently, the dental use of fluoride has received additional negative attention in more recent years as the associations between fluoride exposure and specific types of neurological disorders have become amplified in the online and social media environment—despite the fact that many of these links may be due to endogenous fluoride in groundwater and may not be the direct or indirect result of dental use or treatments [68,69,70].

Finally, this study also revealed significant declines in preventive dental treatment using SDF among this pediatric patient population. As a relatively inexpensive treatment that provides immediate and long-lasting effects, SDF has been the subject of increasing dental public health interest in recent years—particularly among low-income and minority patient populations that might experience barriers and challenges in accessing quality, affordable oral and dental care [71,72]. However, despite these advantages and benefits, there have been limitations regarding the acceptability and use of SDF due to the black staining following application of SDF that has negatively influenced parental perceptions and acceptance of this dental treatment particularly among older children in school who may experience negative social and peer feedback based upon the appearance of SDF-treated teeth [73,74,75]. Although many studies have demonstrated the clinical effectiveness and cost savings of preventive treatments with SDF among children with rampant and severe childhood caries compared with other preventive measures, many parental perceptions regarding the negative aspects of post-application appearance and aesthetics remain significant challenges for oral healthcare providers and researchers [76,77,78].

Despite the significance of these findings, some consideration should also be given to the limitations of this type of retrospective health informatics study, which may limit the inferences that can be made to larger patient populations. For example, these retrospective data were derived from a public dental school clinic that serves a predominantly low-income, minority patient population that relies heavily upon public-funded assistance programs including Medicaid and the Children’s Health Insurance Program (CHIP) [35,36]. Therefore, this specific population of patients and their parents or guardians may not be representative of the general patient population and may be further influenced by other factors including comparatively lower levels of health literacy, lower socioeconomic status (SES), and other significant challenges and barriers to health information, such as limited English proficiency and lower levels of parental education [35,36,37,38]. Moreover, other confounding variables, such as comorbidities and underlying health conditions, were not evaluated, although it is possible that these could have influenced healthcare usage and utilization among this patient population [35,36,37,38]. Furthermore, due to the targeted focus of this initial pilot study, additional factors that may have confounding influences on parental and patient behaviors, such as mental health status, dental anxiety, as well as social media and internet usage, were not explored but could be the subject of further investigation in future prospective studies on this topic [79,80].

## 5. Conclusions

In summary, this study used targeted health informatics to reveal significant changes and shifts in preventive dental services and treatments over time among this pediatric patient population following the COVID-19 pandemic, which may be of interest to public and oral health researchers and other dental providers as they assess the potential factors that drive these changes in pediatric treatment patterns and behaviors among larger populations over time. More specifically, this study demonstrated that significant divergent trends were found following the COVID-19 pandemic for specific preventive measures related to pediatric dental and oral health, with observed increases in the utilization of some longer-lasting treatments (dental sealants) and decreased usage of other preventive services (fluoride varnish and SDF). An awareness and understanding of these shifts in behavior may help planning and outreach efforts to address the current and future needs of pediatric patients and their parents.

## Figures and Tables

**Table 1:** Pediatric clinic population demographics.

Demographic	Pediatric Clinic Population	Statistical Analysis
*Sex*		X^2^ = 0.968, d.f. = 1 *p* = 0.3251
Males	Males (*n* = 11,702/24,460) 47.8%	
Females	Females (*n* = 12,758/24,460) 52.2%	
*Race or Ethnicity*		X^2^ = 454.45, d.f. = 4 *p* = 0.0001
White (Caucasian)	White (*n* = 5576/24,460) 22.8%	
Hispanic (Latino)	Hispanic (*n* = 14,529/24,460) 59.4%	
African American (Black)	Black (*n* = 3229/24,460) 13.2%	
Asian/Pacific Islander	Asian (*n* = 832/24,460) 3.4%	
Other	Other (*n* = 294/24,460) 1.2%	
*Age*		
Average	Average: 9.04 years, ±4.86	
Range	Range: 0–18 years	

**Table 2:** Demographic characteristics of patients receiving preventive services pre- and post-pandemic.

	Dental Sealants	Fluoride Varnish	Silver Diamine Fluoride (SDF)
Age	Age	Age	Age
2017 to 2019 (pre)	Average (pre): 10.51 yrs.	Average (pre): 8.55 yrs.	Average (pre): 6.76 yrs.
2020 to 2022 (post)	Average (post): 10.96 yrs.	Average (post): 8.97 yrs.	Average (post): 6.34 yrs.
Two-tailed *t*-test	*p* = 0.9224	*p* = 0.231	*p* = 0.266
Sex	Sex	Sex	Sex
Male	Male	Male	Male
2017 to 2019 (pre)	Average (pre): 49.9%	Average (pre): 50.3%	Average (pre): 49.9%
2020 to 2022 (post)	Average (post): 50.4%	Average (post): 50.5%	Average (post): 50.3%
Female	Female	Female	Female
2017 to 2019 (pre)	Average (pre): 50.1%	Average (pre): 49.7%	Average (pre): 50.1%
2020 to 2022 (post)	Average (post): 49.6%	Average (post): 49.5%	Average (post): 49.7%
Chi square	*p* = 0.5093	*p* = 0.4911	*p* = 0.511
Treatment number	Treatment number	Treatment number	Treatment number
2017 to 2019 (pre)	2017 to 2019 (pre): 6596	2017 to 2019 (pre): 9730	2017 to 2019 (pre): 1809
2020 to 2022 (post)	2020 to 2022 (post): 8913	2020 to 2022 (post): 7046	2020 to 2022 (post): 1375
Fisher’s exact test	Increase: 35.1%, *p* = 0.012	Decrease: 37.6%, *p* = 0.011	Decrease: 24.0%, *p* = 0.032

## Data Availability

The primary data may be available upon request from the corresponding author. These data are not publicly available according to the protection parameters for the study protocol, which were required by the IRB and OPRS for the study approval.

## Abbreviations

The following abbreviations are used in this manuscript:

ADA	American Dental Association
AAPD	American Academy of Pediatric Dentistry
SECC	severe early childhood caries
SDF	silver diamine fluoride
